# Harnessing an Artificial Intelligence–Based Large Language Model With Personal Health Record Capability for Personalized Information Support in Postsurgery Myocardial Infarction: Descriptive Qualitative Study

**DOI:** 10.2196/68762

**Published:** 2025-04-30

**Authors:** Ting-ting Yang, Hong-xia Zheng, Sha Cao, Mei-ling Jing, Ju Hu, Yan Zuo, Qing-yong Chen, Jian-jun Zhang

**Affiliations:** 1 Department of Cardiology West China Hospital, Sichuan University Chengdu China; 2 Department of Gynecology and Obstetrics Nursing West China Second University Hospital, Sichuan University Chengdu China; 3 West China School of Nursing West China School of Nursing, Sichuan University Chengdu China; 4 Department of Gynecology and Obstetrics West China Second University Hospital, Sichuan University Chengdu China; 5 Key Laboratory of Birth Defects and Related Diseases of Women and Children (Sichuan University) Ministry of Education Chengdu China

**Keywords:** myocardial infarction, post-surgery recovery, personalized health support, artificial intelligence, large language model, personal health record, digital health tools, health information accessibility, qualitative study, mobile phone

## Abstract

**Background:**

Myocardial infarction (MI) remains a leading cause of morbidity and mortality worldwide. Although postsurgical cardiac interventions have improved survival rates, effective management during recovery remains challenging. Traditional informational support systems often provide generic guidance that does not account for individualized medical histories or psychosocial factors. Recently, artificial intelligence (AI)–based large language models (LLM) tools have emerged as promising interventions to deliver personalized health information to post-MI patients.

**Objective:**

We aim to explore the user experiences and perceptions of an AI-based LLM tool (iflyhealth) with integrated personal health record functionality in post-MI care, assess how patients and their family members engaged with the tool during recovery, identify the perceived benefits and challenges of using the technology, and to understand the factors promoting or hindering continued use.

**Methods:**

A purposive sample of 20 participants (12 users and 8 nonusers) who underwent MI surgery within the previous 6 months was recruited between July and August 2024. Data were collected through semistructured, face-to-face interviews conducted in a private setting, using an interview guide to address participants’ first impressions, usage patterns, and reasons for adoption or nonadoption of the iflyhealth app. The interviews were audio-recorded, transcribed verbatim, and analyzed using Colaizzi method.

**Results:**

Four key themes revealed included: (1) participants’ experiences varied based on digital literacy, prior exposure to health technologies, and individual recovery needs; (2) users appreciated the app’s enhanced accessibility to professional health information, personalized advice tailored to their clinical conditions, and the tool’s responsiveness to health status changes; (3) challenges such as difficulties with digital literacy, usability concerns, and data privacy issues were significant barriers; and (4) nonusers and those who discontinued use primarily cited complexity of the interface and perceived limited relevance of the advice as major deterrents.

**Conclusions:**

iflyhealth, an LLM AI app with a built-in personal health record functionality, shows significant potential in assisting post-MI patients. The main benefits reported by iflyhealth users include improved access to personalized health information and an enhanced ability to respond to changing health conditions. However, challenges such as digital literacy, usability, and privacy and security concerns persist. Overcoming the barriers may further enhance the use of the iflyhealth app, which can play an important role in patient-centered, personalized post-MI management.

## Introduction

Myocardial infarction (MI) remains a leading global cause of morbidity and mortality, despite significant advances in surgical and clinical interventions [1,2]. While improved medical care has increased survival rates, effective postsurgical recovery management has become increasingly essential [3,4]. Patients recovering from MI surgeries frequently encounter diverse challenges, including persistent symptoms, difficulties with medication adherence, necessary lifestyle adjustments, and psychological stress such as anxiety and depression [5-7].

Current post-MI recovery support primarily relies on generic educational materials or standard clinical consultations. Such conventional methods often lack sufficient customization to address individual patients’ unique medical and psychosocial contexts, resulting in information that can feel irrelevant or insufficient [8,9]. Personalized information delivery has demonstrated substantial benefits, including improved medication adherence, better symptom management, reduced anxiety, and increased patient confidence, ultimately leading to better recovery outcomes and lower hospital readmission rates [10-12]. However, achieving personalized information delivery at scale remains challenging due to resource constraints and the limitations of traditional health care delivery methods.

Advances in artificial intelligence (AI), particularly large language models (LLMs), have opened promising avenues for delivering personalized health information. LLMs leverage natural language processing to provide sophisticated, human-like communication, accurately understanding complex medical queries and generating detailed, context-specific responses [13,14]. Yet, most existing LLM-based AI health tools offer only general health guidance unless patients manually input health information during interactions, limiting their effectiveness in managing chronic conditions or complex recoveries, such as post-MI care [15].

Integrating LLMs with personal health records (PHRs) represents a novel approach to overcome this limitation. PHRs are electronic apps enabling users to securely maintain and manage comprehensive personal health data, such as medical history, medications, laboratory results, and allergies. When combined with LLMs, these tools can automatically generate personalized responses tailored explicitly to each user’s recorded health context [15,16]. However, despite the clear potential of AI-PHR integration, literature regarding their practical application and patient experiences, particularly in post-MI care settings, remains sparse.

This study aims to address this gap by investigating the user experiences and perspectives of post-MI patients regarding an innovative LLM AI health tool, iflyhealth, which includes an integrated PHR module. By exploring real-world patient interactions with this tool, we seek to identify its supportive capacities, assess barriers and facilitators influencing user engagement, and understand how such technology might be best leveraged to support individualized post-MI recovery. Insights from this research could contribute significantly to the design and implementation of future patient-centered digital health interventions, facilitating improved health outcomes through tailored AI-based information support.

## Methods

### About the LLM AI Tool

**Figure 1:**
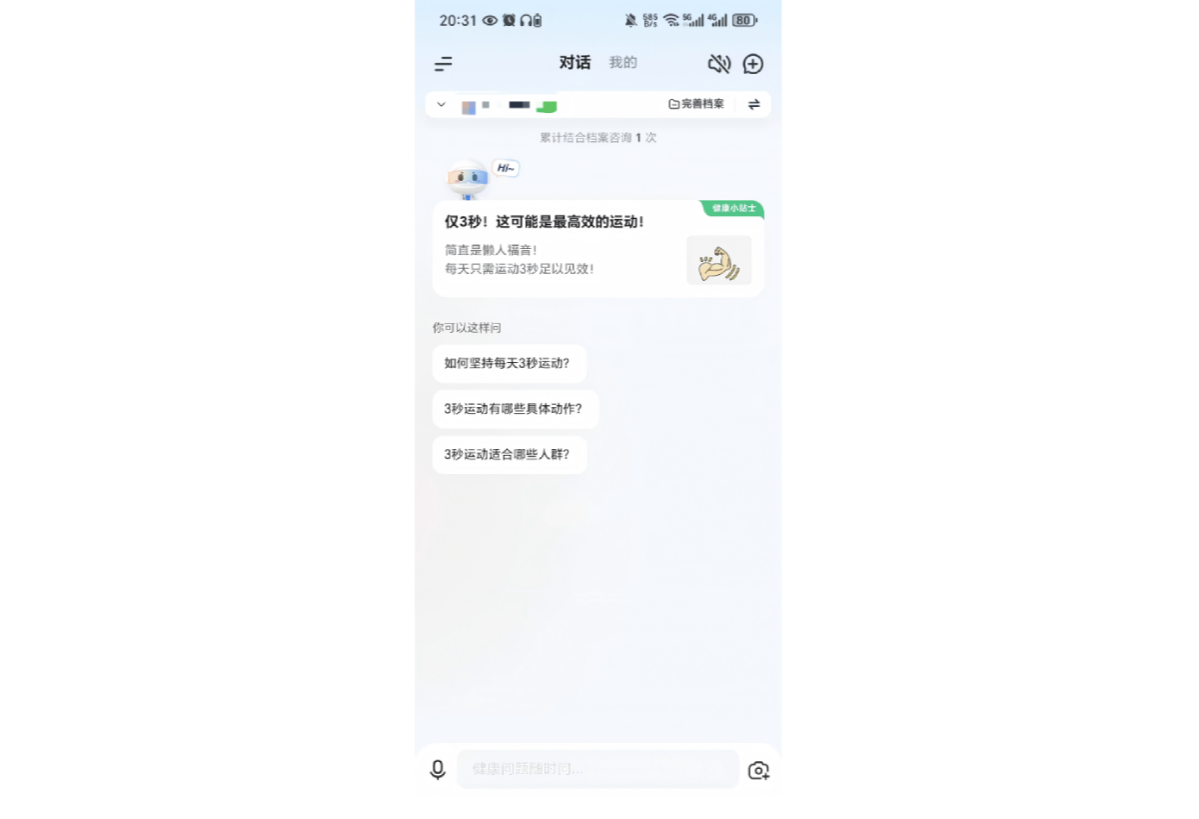
User interface of the iflyhealth app: chatbot. Reproduced with permission from iflyhealth.

**Figure 2:**
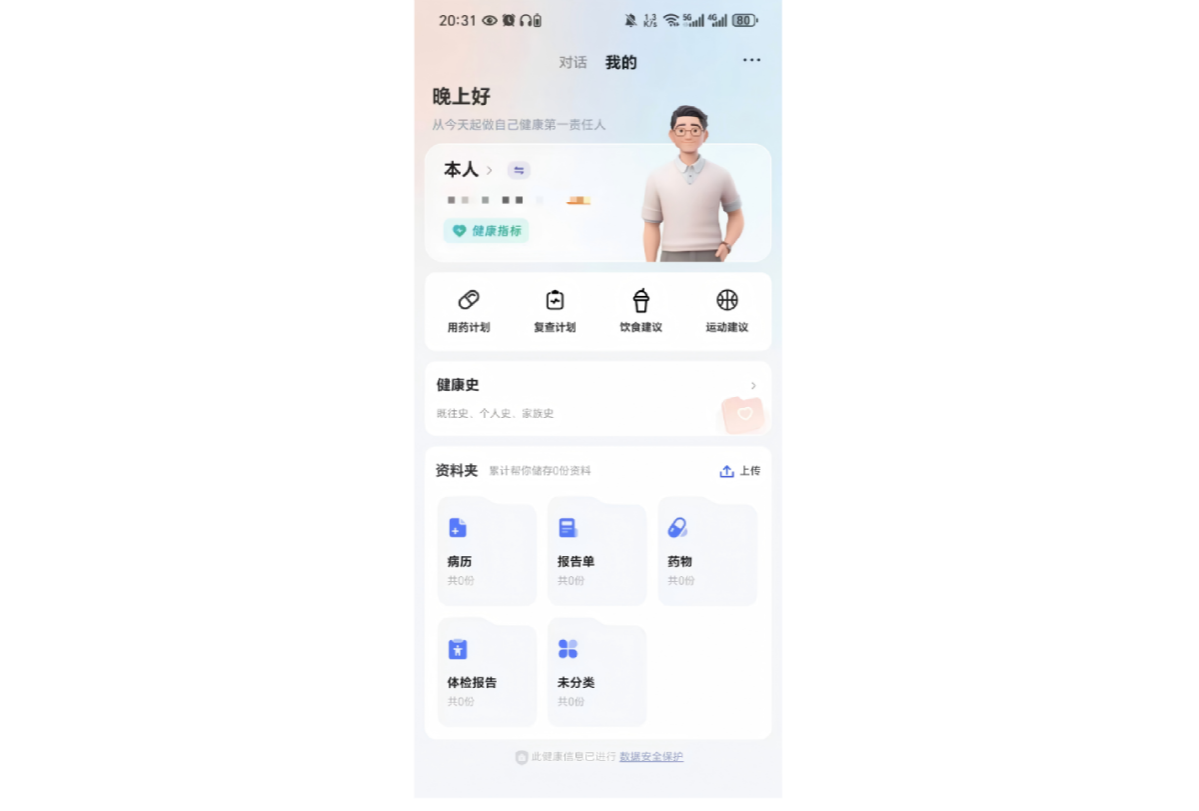
User interface of the iflyhealth app: personal health record module. Reproduced with permission from iflyhealth.

**Figure 3:**
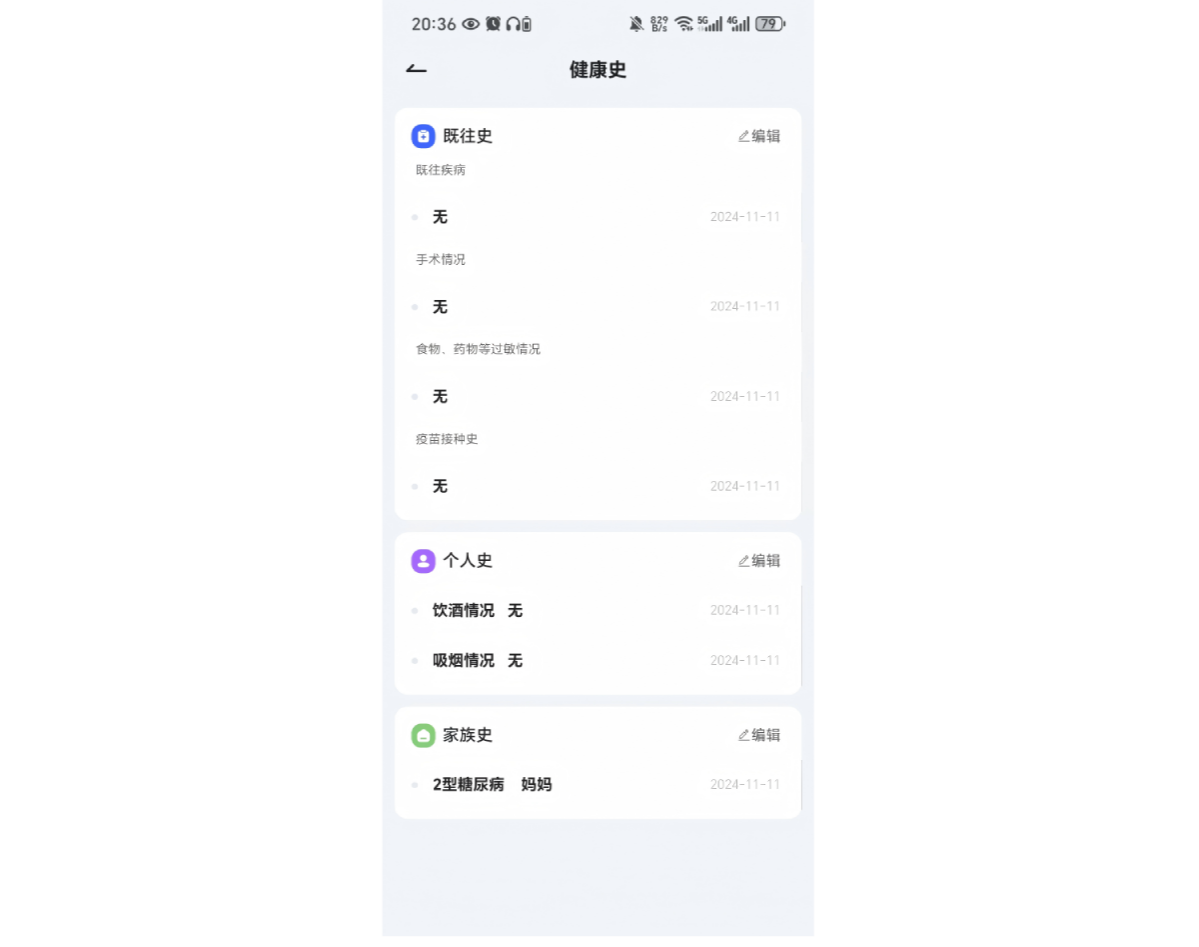
User interface of the iflyhealth app: health history module of the personal health record module. Reproduced with permission from iflyhealth.

The iflyhealth (讯飞晓医) app is developed by iFLYTEK [17]. It is an AI-powered health chatbot designed to provide health-related information. The app offers various health management capabilities, including symptom checking, disease diagnosis, medication guidance, and lifestyle recommendations. Notably, the app is available only in Chinese. Figure 1 shows the chatbot interface of iflyhealth. What sets iflyhealth apart from similar apps is its integrated PHR module. This module allows users to enter and save a wide range of detailed health information, such as their medical history, medications, allergies, and family health history. The app also enables users to upload images of laboratory tests, checkup reports, and other documents to receive personalized insights from the AI (Figures 2 and 3). The English translation of the Chinese text and feature descriptions in the interfaces captured in Figures 1-3 are provided in Multimedia Appendix 1.

When selecting an AI tool for recommending to the patients on our patient support program, we conducted an empirical evaluation of available LLM AI apps at the time. We began by compiling a list of candidate apps and then assessed each based on the following criteria: the ability to provide high-quality, clinically relevant information; flexibility in offering tailored care; and cost-free access for users. We also examined usability factors such as interface design and ease of navigation through preliminary trials with a small group of potential users. iflyhealth emerged as the best candidate because of its integrated PHR module, besides other features. The PHR module allowed users to record detailed health information and upload report files, and provide health information using a more focused medical knowledge base compared with other LLM AI apps. Its free availability further ensured broad dissemination among patients without financial constraints.

### Study Setting and Design

The research was carried out in the Department of Cardiology at West China Hospital, Sichuan University, one of the largest cardiology centers in Southwest China. Our department offers high-volume tertiary care for adults with cardiac disease. In March 2024, we introduced the iflyhealth app into our postsurgery patient support program for patients who had undergone MI surgeries in the previous 6 months, to provide personalized information support for recovery and health maintenance.

When introducing the app to patients, a nurse typically approached them during their hospital stay after admission and briefly explained how the iflyhealth app works and its potential as a complementary tool to formal consultations. The nurse provided a short demonstration on using the app, particularly the PHR module, once patients downloaded it onto their smartphones. Additionally, explicit instructions were given regarding the possibility of errors in the app’s responses, the importance of not relying solely on the app for health-related information, the precedence of clinicians’ advice, and the need to seek care in case of emergencies.

We used a descriptive qualitative study design to gain an in-depth understanding of patients’ experiences with iflyhealth during postsurgical recovery and to explore why some patients refused or discontinued using the tool. This approach is better suited for our study than other qualitative methods because it allows us to capture and present concrete, context-specific details about user experiences without requiring the development of abstract theories. While phenomenological approaches focus on uncovering the underlying essence of lived experiences, our primary goal was to document practical insights and real-world perceptions regarding the tool’s impact on personalized health information. Given that iflyhealth is a newly emerging technology, the descriptive qualitative approach enabled us to directly record detailed patient perspectives and experiences in a straightforward manner that is immediately applicable to clinical practice and future research [18].

### Sampling

We used purposive sampling to select participants from our postsurgery support program [19]. This included individuals who had used the iflyhealth app during their recovery and those who had refused or stopped using it. The inclusion criteria were adult patients (aged 18 years or older) who underwent MI surgery in the last 6 months, used the iflyhealth app at least 3 times (users) or refused to use or discontinued its use (nonusers), had no known psychological or cognitive conditions that could influence this study’s findings, possessed adequate communication abilities, and were able to give informed consent. Patients were excluded if they were aged younger than 18 years, had psychological or cognitive conditions, faced communication barriers, or were unable to provide consent. To ensure participant diversity, we recruited individuals across different age groups, social statuses, education levels, and usage patterns of the iflyhealth app (including both users and nonusers) to capture a broad range of feedback.

### Data Collection and Analysis

We collected data through face-to-face semistructured interviews [20] to understand patients’ experiences and perceptions regarding the use or nonuse of the AI tool for postsurgery information support. We developed an interview guide to ensure that all relevant topics were addressed (see Multimedia Appendix 2 for the interview guide) [21]. The guide included open-ended questions about participants’ initial impressions and expectations of the tool, their experiences using it for daily health management, the difficulties they faced, and the reasons for refusing or discontinuing its use. Probing questions were included to explore certain areas in greater depth, while maintaining flexibility based on participants’ responses. The initial version of the interview guide was pilot-tested with 3 participants. The feedback was used to revise the interview guide. The piloting participants’ data were not included in the subsequent formal analysis.

Interviews were conducted in a small, quiet meeting room in the cardiology department to preserve confidentiality and encourage open discussion. We used techniques such as active listening, probing, silence, and paraphrasing to thoroughly explore each participant’s perspectives and experiences. We requested permission to audio-record the interviews, and the recordings were transcribed verbatim shortly after the interviews to prevent data loss. To ensure confidentiality, all identifying information was anonymized during transcription. Data collection continued until no new meaningful information emerged in 2 consecutive interviews, indicating that data saturation had been reached.

We analyzed the data using Colaizzi data analysis method [22]. Thematic analysis is particularly suited for our study because it enables a systematic exploration and interpretation of the nuanced, multifaceted experiences of post-MI patients using the iflyhealth app, capturing both common patterns and individual variations in their recovery journeys. Coupled with a descriptive qualitative research approach, this method is especially appropriate for investigating the use of the iflyhealth app, a newly emerging technology, in conventional health information–seeking settings.

In analyzing the data, initially, we independently read the interview transcripts 2 to 3 times to fully immerse ourselves in the data and understand each participant’s narrative. Significant statements directly reflecting experiences and perspectives related to the app were then extracted, especially initial impressions, usage patterns, perceived benefits, and encountered challenges. The statements were then analyzed to formulate underlying meanings, which were systematically coded to reflect both the explicit content and contextual nuances. Similar codes were then grouped into subthemes, which were further clustered into main themes to develop an integrated description of the participants’ experiences. Finally, the preliminary findings were validated through member-checking with participants to ensure that our interpretations accurately reflected their experiences and perspectives.

### Study Rigor

To ensure study rigor, we used 4 methods to address credibility, transferability, dependability, and confirmability [23-25]. Repeated reading and in-depth discussion of the data enhanced credibility. Only 1 interviewer conducted all interviews (except for 3 interviews conducted by another researcher due to the initial interviewer’s involvement in the participant’s treatment team), ensuring consistency in the interviewing process. Member-checking allowed participants to verify the accuracy of our findings and confirm that our interpretations aligned with their views. Detailed descriptions of this study’s setting, participant characteristics, and context were maintained to improve the transferability of our work. A study journal documented the audit trail for each step of data collection and analysis, and it included personal reflections on the research process to enhance confirmability. Regular research team meetings and peer debriefing sessions were held to ensure that our analysis and interpretations were firmly grounded in the data.

### Ethical Considerations

Our study was ethically approved by the Ethics Committee of West China Hospital, Sichuan University (20241638). We explained this study’s purpose, procedures, and the participants’ right to withdraw at any time without penalty before enrolling each participant. Written informed consent was obtained from all participants before their enrollment. This study was conducted per the Helsinki Declaration and other applicable ethical standards. Identifying information in the demographics and interview transcripts was either removed or replaced with participant codes. All electronic data were securely stored on a password-protected flash drive kept in a locked drawer in the first author’s office, along with any paper materials such as field notes. Access to the data was strictly limited to the research team and was used solely for the purposes of this study. Participants were informed about the privacy and confidentiality protection measures before the interviews. No compensation was provided for participation, and no photos or other images were collected or used in this study.

## Results

### Participant Demographics

We interviewed 20 participants, including 12 users and 8 nonusers, from July 9 to August 19, 2024 (Table 1). The interviews lasted for 19-37 (average 23.9) minutes. The user group was slightly younger, with most participants in their 50s, while nonusers were mostly in their 60s. Users were nearly evenly divided between males and females, whereas nonusers were predominantly male. The users tended to have a higher education level, with most holding bachelor’s or graduate degrees. In contrast, more than half of the nonusers had a high school education. Their employment statuses also differed. Most users were still working, while two-thirds of the nonusers were retired. The average time since surgery at the time of the interview was similar for both groups, and most participants in each group were married.

**Table 1:** Participant characteristics (N=20).

Characteristic		Users (n=12)	Nonusers (n=8)
**Age (years)**			
	Mean (SD)	49.3 (12.2)	53.7 (12.5)
	Range	29-69	32-75
**Gender, n**			
	Female	5	2
	Male	7	6
**Education level, n**			
	High school or below	2	5
	Bachelor’s degree	7	3
	Postgraduate degree	3	0
**Employment, n**			
	Employed	8	2
	Retired	4	6
Time since MI surgery (months), mean (SD)		3.5 (1.2)	3.7 (1.5)
**Marital status, n**			
	Not married	3	3
	Married	9	5

### Themes

**Figure 4:**
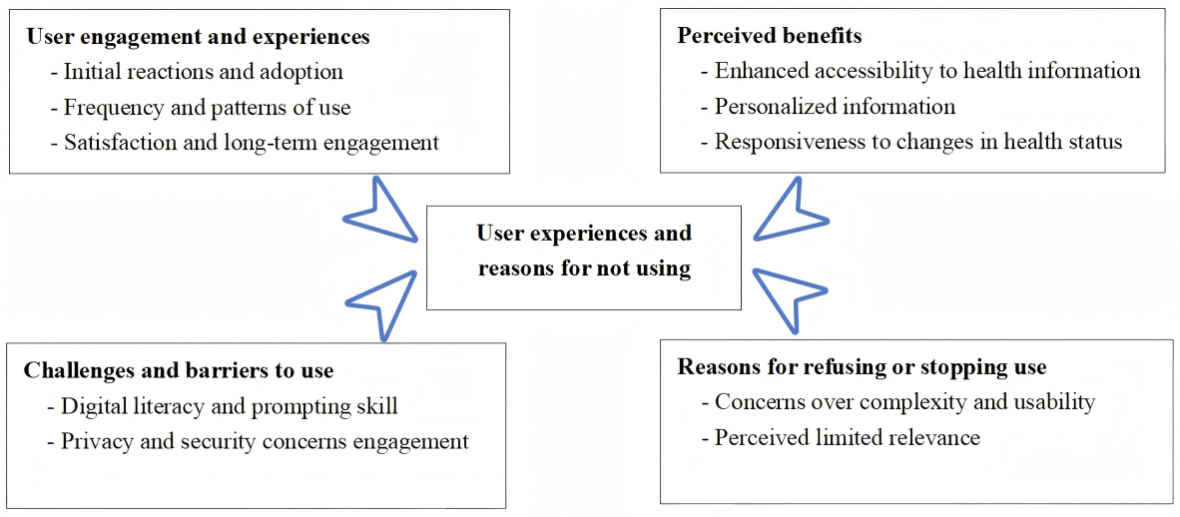
Key themes about user experiences and reasons for not using the LLM AI tool. AI: artificial intelligence; LLM: large language model.

A total of 4 themes with 10 subthemes were revealed (Figure 4).

### Theme 1: User Engagement and Experiences

#### Overview

Participants engaged with iflyhealth in ways that varied substantially based on their experience with digital health technologies, personal health needs, and perceptions of the tool’s value. Some found it a useful supplementary resource during their recovery, while others used it less frequently. Long-term engagement was influenced by satisfaction. Participants who found the tool relevant and easy to use tended to continue using it.

#### Subtheme 1.1: Initial Reactions and Adoption

Participants had varied expectations and reactions depending on their familiarity with digital health tools. For some, the tool appeared to be a promising addition to support their recovery, while others were skeptical or reluctant (Textbox 1).

**Initial reactions and adoption.**

When first hearing about the AI (iflyhealth), I was really curious. I thought it could be a good method to help with my recovery after I received the surgery... Still a bit skeptical, too. I heard others saying that these health apps don’t really do much, so I wasn’t sure if it’d actually be helpful.

P4

I felt interested and curious when you (nurse) first introduced me to the app. I have always been keen on keeping up with technological advances. The AI sounded like a smart tool, especially when you said that it could give advice according to my own conditions after surgery. It took some time to figure out how to use it. It was still a little difficult as a new technology and I was a first-time user. I enjoy using it after I learned how.

P9

To be honest, I was doubtful at first. I thought if I had questions, I could just ask the doctor. But they assured me it was simple to use, though it was initially difficult to figure out on my own. Later, it got better.

P10

#### Subtheme 1.2: Frequency and Patterns of Use

Participants used the app in different ways. Some used it regularly in their daily routines, while others used it sporadically based on their health needs. The usage patterns were influenced by factors such as the perceived relevance of the answers and the ease of navigating the app’s features:

I try to use it every day, especially when I want to look up something. It is much easier than searching the Internet. It is much better than waiting for the next time I see the doctor. It’s like a habit now. I even ask in the morning sometimes about what I should eat and drink for breakfast.

P2

I don’t use it often. Mostly, I check it when something comes up, like if I feel a new symptom or have a question about my medication. It’s more like a backup.

P4

Not regularly. It depends on how I feel. If I’m feeling fine, I might just forget about it. When I feel unsure or need advice but cannot see a doctor, I use it.

P11

#### Subtheme 1.3: Satisfaction and Long-Term Engagement

Satisfaction with the tool’s functions influenced long-term use. Some users relied on it to manage their daily health, appreciating its accessibility and the quality of information provided. Others stopped using it or lost interest because the app did not fully meet their needs or because the answers were not sufficiently relevant:

I’ve kept using it because it indeed makes things easier, especially once you know how to ask it questions. Its answers become really helpful, especially when it provides advice specific to my conditions.

P2

Personally, it’s been really helpful. It feels like having a doctor always available to answer questions. Sometimes the answers are not so accurate or are too broad. I ask this, but it answers that. However, overall it is much better than having no one to answer my questions. Most of the answers are still good... I will continue using it and even suggest it to others.

P7

I used it several times after discharge, then stopped using it much. It was okay, but I didn’t feel like it offered much beyond what I already knew. Maybe it works better for others, but it did not provide what I needed.

P11

### Theme 2: Perceived Benefits

#### Overview

Participants recognized many advantages of the tool, particularly its accessibility, personalized health information, and its ability to adjust responses based on health changes. The app was seen as a reliable information source that provided a sense of control and confidence:

#### Subtheme 2.1: Enhanced Accessibility to Health Information

Participants found that having access to health information at any time was very valuable for answering day-to-day questions. They felt the app provided reassurance when navigating health concerns because it was easily accessible and helped them understand complex health concepts (Textbox 2).

**Enhanced accessibility to health information.**

Always usable, that’s the biggest advantage. More important than the answers themselves.

P2

Usable anytime, saves time, and the answers are professional. Instead of searching the Internet and worrying about accuracy or encountering scam information, I can just open the app and ask... It is very reassuring.

P9

Better than bothering my family or going to see a doctor. It is like having a personal doctor who can answer questions at all times... I cannot tell how good the answers are, since I’m not a doctor, but the tone sounds very professional... The key is it is very convenient, and it can answer according to my conditions.

P10

One is quick; two is accurate; also, it has no temper, unlike a doctor. I can keep asking. It always answers... I can get answers about my recovery right away... It’s like having a small doctor in my pocket. As soon as I need it, I can ask.

P12

#### Subtheme 2.2: Personalized Information

Participants noted that iflyhealth delivered personalized information based on their specific health history and postsurgery needs. This personalization made the responses more relevant and supportive for their recovery:

After I learned how to use the health information (PHR) feature, it really felt like it understood my conditions. Its answers became more specific, especially after I entered my information.

P4

Specific (personalized) advice is, of course, more helpful. Initially, it was generic like what you find online, but after entering my information, it became more tailored to my conditions.

P9

This AI is different because it can recognize my situation. With other tools, I must tell the AI every time I talk with it. The answers are more specific to my situation... Sometimes the answers are still generic, but if I ask in a different way, they become better.

P11

#### Subtheme 2.3: Responsiveness to Changes in Health Status

Participants appreciated the AI tool’s ability to adapt to changes in their health status. Some reported that it provided new recommendations based on updated health information, which helped the responses better match their recovery process:

When I had new symptoms, I would update the app, and the AI would give me advice based on those changes. This is particularly good.

P4

It can adapt to the changes I enter and provide different advice, for example, after I adjusted my medication or experienced new symptoms or received a new exam report. I did not expect the AI to be so advanced nowadays.

P6

Being able to update the answers with my new information takes it to another level.

P10

### Theme 3: Challenges and Barriers to Use

#### Overview

Despite its advantages, participants faced several barriers that prevented them from using or continuing to use the tool, including technical issues such as difficulties in finding needed features or understanding the app’s functions, as well as concerns about data privacy. Limited digital literacy also resulted in a lower comfort level with using the app, reducing the willingness to continue its use.

#### Subtheme 3.1: Digital Literacy and Prompting Skill

Participants’ digital literacy significantly impacted their experience. Those with less experience using digital tools required more guidance to use the app properly. Some reported difficulties in formulating prompts for the AI, which led to less useful responses (Textbox 3).

**Digital literacy and prompting skill.**

I’m not used to using apps, not even using a cell phone, so figuring out the AI was really difficult at first. It took me two visits to the hospital to really learn how to use it... My son helped me too.

P7

In the beginning, it was very difficult to get the answers I needed because I didn’t know how to use it. I’m never good with electronic devices. I almost gave up... You (nurse) taught me for a long time before discharge. My son learned it and taught me again after discharge... It remained difficult until I became familiar with it, especially when I couldn’t see the doctor right away.

P8

I’m a tech-idiot. But I was interested when you (nurse) mentioned that it could answer questions outside the hospital, so I took the time to learn how to use it... It became easier the more I used it.

P12

#### Subtheme 3.2: Privacy and Security Concerns

Some participants expressed concerns about data privacy. They questioned whether their personal health information would be secure on a digital platform, which directly affected their willingness to enter information and impacted adoption and the tool’s ability to provide personalized recommendations:

I wasn’t sure how secure my information would be, so I didn’t enter personal details. I have heard a lot about data breaches.

P13

At first, I really had doubts. It involved my personal health information after all. But later, I learned that I did not have to reveal my identity, and I felt fine. The answers became better with the (added) information.

P9

Privacy felt like an issue. I felt uncomfortable entering all my health history on an app. Who knows how the company might use my information.

P14

### Theme 4: Reasons for Refusing or Stopping Use

#### Overview

Some participants did not use the tool at all after discharge, while others started but soon discontinued use due to difficulties such as outdated devices, complexity, or lack of relevance. The reasons for nonuse varied from challenges during the initial use to a preference for traditional methods. Participants who stopped using the app later reported that the responses were not always specific or relevant to their needs.

#### Subtheme 4.1: Concerns Over Complexity and Usability

Some nonusers reported issues such as having outdated technology or technology that is not a smartphone, low confidence in using new technology, and difficulty learning how to use the app. Feedback indicated that usability issues and a complicated, inaccessible interface discouraged further use:

My phone was too old, and it doesn’t support apps like this.

P15

I’m not very good at technology. When I first opened it, I found it confusing... I couldn’t understand it when you (nurse) taught me.

P16

Too complicated. Perhaps something simpler would work for me... It could take a lot of time to learn how to use it. Too much trouble.

P18

I tried a couple of times but couldn’t figure out how to use the personal advice feature. I couldn’t get used to it, so I gave up.

P20

#### Subtheme 4.2: Perceived Limited Relevance

Some participants felt that the answers and recommendations were not sufficiently relevant to their individual needs. They questioned whether the tool could be truly helpful, noting that the advice was too generalized and not specific enough to their conditions, which discouraged continued regular use:

Useful, but the answers are too general. I stopped using it because it kept giving broad responses. Some were useful, and some were not. I could get most answers from doctors too, so I didn’t continue using it.

P13

I wanted something more focused on my conditions, but it felt too generic, so I stopped using it.

P17

## Discussion

### Principal Findings

In this study, we investigated the user experiences and perspectives of patients after MI surgeries regarding an AI-based LLM tool with a built-in PHR. Our findings demonstrate the complexity of user engagement and reveal both the advantages and disadvantages of adopting such an emerging AI-based health tool for supporting health information during recovery.

While recognizing the strengths and benefits of iflyhealth with a built-in PHR module for delivering timely, accessible, and personalized health information, participants also reported usability issues with the iflyhealth app. For example, some pointed to specific design flaws that hindered their experience. An example was how to activate the PHR module. The icon to activate it was small and unintuitive. Users could either overlook the icon or forget to tap it to turn on the PHR for personalized responses. This resulted in sessions without personalized content. This usability issue indicates a discoverability problem, which is that a key functionality is essentially hidden in the interface. Prior studies emphasize that poor interface usability can directly lead to user frustration and abandonment [26-28]. If core features are not easily accessible or visible, users may not be able to realize the tool’s full benefits, as seen in our study when personalized recommendations were missed. Our findings align with broader usability research showing that when important functions are hard to find, the app’s value diminishes and satisfaction plummets [29,30]. In essence, the iflyhealth app’s design did not adequately support users’ natural navigation habits. A more user-centered design approach, for instance, making the personalized mode a default or clearly prompting users to activate it, may help to mitigate the issue.

Our analysis suggests that sustained engagement with the iflyhealth tool depends on a combination of usability, user skills, personalization, and trust in the information provided. Participants who continued using the app often find it reasonably easy to navigate and useful, whereas those who discontinued often cite obstacles or insufficient perceived value. This reflects a common pattern in digital health, which is that perceived ease of use and relevance are pivotal for long-term adoption [31-33]. If an app is cumbersome or confusing, users are likely to drop out quickly.

Additionally, digital literacy is another important factor. Users with higher comfort in using smartphones and apps are better able to explore the app’s features, such as enabling the PHR module and integrating the tool into their routines. In contrast, those with limited experience in digital tools felt overwhelmed, which is consistent with literature noting that technology illiteracy can limit adoption of eHealth tools, especially among older adults [34]. This suggests some nonusers may have disengaged not because the content lacked merit, but because they lacked the support to use the technology effectively.

Another key factor was personalization and perceived relevance of content. Participants who did enable and use the personalized features reported feeling that the health recommendations fit their needs better, which in turn motivated them to keep using the app. Personalization likely made the content more engaging, as generic tips can feel irrelevant or repetitive over time. This is consistent with evidence that tailored interventions, for example, personalized reminders or feedback, tend to increase user engagement in health apps [35-37]. However, users who used the app in a one-size-fits-all manner, without turning on the PHR personalization, are likely more prone to boredom or indifference. For example, some described the app as not telling them anything new, which illustrates how a lack of personalization can hasten discontinuation.

Finally, trust in the AI-generated information plays a role in sustaining use (or not). Even if an app is easy to use and personalized, users may not continue if they doubt the accuracy or safety of its advice. In our study, trust was a vital factor for many participants, who needed to feel confident that the app’s suggestions were reliable and in line with medical knowledge. Those who trusted the information were willing to return, whereas those who encountered answers they deemed dubious lost interest. This finding is echoed by engagement research, noting that the credibility of content influences whether users stick with a digital health tool [38]. As reflected in our findings, the multifactorial nature of engagement underlines why mHealth (mobile health) interventions frequently struggle with retention. Further research is needed to address usability, support, and trust to promote adoption and long-term use of AI-driven apps such as iflyhealth for personalized health information delivery.

It is noteworthy that there are some demographic differences between the user and nonuser cohorts in our study. In general, younger and more educated participants, as well as those employed in professional occupations, are more likely to adopt the app and use it regularly. Older individuals, retirees, and people with lower education levels are overrepresented among the nonusers. This trend is consistent with the digital divide observed in health care technology adoption, where older age and lower educational attainment are associated with lower usage of digital health tools. Older individuals or those with a lower education level tend to face more barriers in adopting new technologies, often due to a combination of limited digital skills, less access, and different health management habits [39-41].

Digital literacy may be a crucial underlying factor connecting the demographic trends. Those with higher education often had better baseline eHealth literacy. They know how to navigate apps, interpret online information, and troubleshoot basic issues. In contrast, some with lower education or of older age may struggle with installing apps, adjusting settings, or interpreting the app’s health metrics. Notably, health and technology literacy have been identified as central to the success of PHR systems and similar tools [42].

Employment status and lifestyle can also play a role, albeit intertwined with age and education. Those who are employed (especially in urban, information-focused jobs) may have more access to smartphones and the internet and a greater incentive to use tools to save them time or improve productivity, for example, using an app to quickly check a health concern at work. Unemployed or retired individuals, in contrast, might not use smartphone apps as frequently in daily life. Additionally, they tend to have more time for conventional approaches, such as seeing a doctor, instead of relying on an app.

Furthermore, people in professional groups may feel more confident in trying a new health technology, whereas others could worry about making mistakes or feeling “too old” for such apps. These sociodemographic disparities suggest that without targeted strategies, AI health tools such as iflyhealth might primarily attract a relatively privileged subset of users while failing to engage those who could arguably benefit greatly from accessible health guidance, such as older adults with chronic conditions. Therefore, it is imperative to design and promote digital health interventions more inclusively, for instance by tailoring training or support for older users, simplifying interfaces for those with lower tech literacy, and addressing the specific concerns of different demographic groups, such as privacy and safety concerns. However, it must be noted that our findings concerning demographic differences are preliminary as a qualitative study, which should be interpreted and considered with caution. Future quantitative research is necessary to confirm the trends and establish causal relations for guiding practice.

Privacy and data security concerns are a vital theme in our findings, especially among the participants hesitant about or resistant to using the app. Some expressed unease about entering personal health information into an AI-driven app, worrying where that data might end up and who could access it. Some nonusers stated outright that they could not trust an app with sensitive health details, referencing data breaches or unauthorized data sharing in the tech industry. This apprehension had a tangible effect on adoption. This is consistent with findings in the broader literature, where confidentiality and security concerns are consistently cited as major barriers to mHealth adoption [43-45]. Notably, such concerns may be more prominent among certain demographic groups. For example, generational studies show that older adults are significantly concerned about privacy and security in digital health [46,47], though such concerns tend to diminish quickly [48]. Furthermore, trust in the AI’s handling of data can intertwine with trust in its content. If users are unsure if the system is secure, they may also tend to doubt its overall credibility [49,50].

These privacy concerns have implications not just for initial adoption but also for engagement. The shadow of potential data misuse could lead to limited acceptance of the app. From a critical perspective, this highlights a gap between what the technology could offer and what users are willing to accept. No amount of advanced AI functionality will matter if the foundational trust in privacy is not established. Our findings urge developers and health care providers to take such concerns seriously, which echo the literature in suggesting that strong privacy assurances and visible security measures are needed to convince users to attempt or initiate use of such AI tools [51]. Additionally, educating users about how their data is handled, for instance, clarifying that data stays on the device or is anonymized in the cloud, could improve trust [52].

The question of AI reliability and trustworthiness is another critical concern in participants’ evaluations of iflyhealth. While the app’s AI capabilities, such as providing instant answers or personalized tips, were a major strength, many users did not blindly trust the app’s responses. Participants’ concerns about accuracy were evident. For instance, if the app’s suggestion for managing a symptom differs from what their physician had earlier advised, users can grow skeptical. Such caution is echoed by broader public sentiment. Prior research shows wide user distrust in AI-driven medical advice on its own. Instead, many still prefer human doctors for critical decisions, or will trust AI only when it is endorsed or monitored by health care professionals [53,54]. Our participants also exhibited this split trust, who are intrigued by the AI’s potential but not ready to rely on it unquestioningly.

Transparency and veracity of information are paramount for AI tools in health. When the AI provides answers not consistent with known medical information, users’ trust in the system may grow. When it produces an answer that seems odd or incorrect, trust can be undermined quickly. Current literature on trust in medical AI identifies perceived accuracy, reliability, and safety of the AI as key drivers of user acceptance [55]. Users in our study explicitly voiced such concerns, which reflect real issues that have been noted in other contexts. The broader implication is that for AI-based health tools, gaining user trust requires more than just technical accuracy. It requires user perception of accuracy and fairness. Participants in our study are essentially calibrating their trust based on every interaction. Additionally, trust can also be transferred from external validation, for example, if a doctor or nurse recommends the app or confirms its advice, the user may feel more confident in using it. This suggests a potential pathway to improving trust, which is integrating AI tools with clinical endorsement or oversight.

### Implications for Practice

Our findings may have several important implications for the design and implementation of AI-assisted health tools and clinical practice. First, addressing digital literacy barriers is essential. Providing targeted user education through digital tutorials, step-by-step guides, and contextual help can improve usability and ensure that even less technology-savvy patients can navigate the tool effectively. Second, robust privacy and security measures are crucial for building trust. Transparent communication about data usage policies, strict adherence to data protection laws, and involving users early in the design process can help mitigate privacy concerns. Third, the personalization features, including the PHR module, can be further enhanced by addressing individual preferences and improving the app interface. Our findings can guide the design of future AI tools and the development of training programs for both patients and clinicians, ensuring that digital health technologies are accessible, secure, and tailored to meet the needs of diverse user groups. Clinically, integrating AI tools such as iflyhealth into routine care might support patient self-management, reduce the burden on health care providers by offering timely and personalized advice, and complement formal consultations. Additionally, for assisting patients and clinicians adopting the iflyhealth app, we are working to create a toolkit based on our findings and experience.

### Limitations

This study has 2 main limitations. First, as a qualitative study conducted at a single tertiary care center, our findings may be context-specific and not fully transferable to other settings or populations. The experiences of post-MI patients using the iflyhealth app in our region might differ from those in other regions or health care systems, where specific research may be needed. Our findings may serve as a reference. Second, although our sample size was sufficient to achieve data saturation in this qualitative study, it does not support the establishment of correlations or allow for detailed comparisons between groups. Future quantitative studies with larger samples are necessary to confirm some of the preliminary findings of our study, such as the differences in gender, age, education level, and employment status between users and nonusers.

### Conclusions

iflyhealth, an LLM AI app with a built-in PHR functionality, shows significant potential in assisting post-MI patients. The main benefits reported by iflyhealth users include improved access to personalized health information and an enhanced ability to respond to changing health conditions. However, challenges such as digital literacy, usability, and privacy and security concerns persist. Overcoming the barriers may further enhance the use of the iflyhealth app, which can play an important role in patient-centered, personalized post-MI management.

## Appendix

Multimedia Appendix 1 Translation of text and feature descriptions in figures.

Multimedia Appendix 2 Interview guide (Chinese original and English translation).

## Abbreviations

AI: artificial intelligence
LLM: large language model
mHealth: mobile health
MI: myocardial infarction
PHR: personal health record

## Data Availability

In accordance with institutional privacy policy requirements, the audio recordings of the interviews cannot be shared. Desensitized demographic data and interview transcripts are available from the corresponding author upon reasonable request.
