# Supplementary material for: Harnessing an Artificial Intelligence–Based Large Language Model With Personal Health Record Capability for Personalized Information Support in Postsurgery Myocardial Infarction: Descriptive Qualitative Study
Source: J Med Internet Res. 2025 Apr 30;27:e68762. doi: 10.2196/68762 (PMC12079068; doi:10.2196/68762)
Supplement: Multimedia Appendix 1 [file jmir_v27i1e68762_app1.pdf]

Appendix 1: Translation of text and feature descriptions in Figures 1-3

Figure 1
对话: Dialogue
Feature description: Chatbot mode interface.

我的: My
Feature description: Switch to Personal Health Record (PHR) interface.

完善档案: Complete PHR
Feature description: Enter PHR data.

结合档案咨询 1 次: 1 consultation with PHR data integrated
Feature description: Count of consultations with PHR data integrated.

仅 3 秒!这可能是最高效的运动!
健康小贴士
简直是懒人福音!
每天只需运动 3 秒足以见效!
Just 3 seconds! This could be the most efficient exercise!
Health tips
Great news for the lazy!
It only takes 3 second a day to see effects!
Feature description: Health tips posts provided by the app.

你可以这样问
如何坚持每天 3 秒运动?
3 秒运动有哪些具体动作?
3 秒运动适合哪些人群?
You can ask like this
How to stick to 3 seconds of exercise a day?
What are the movements of a 3-second exercise?
Which people is the 3-second exercise suitable for?
Feature description: Suggested questions. Users may click on the questions, saving them from phrasing their own questions.

健康问题随时问...
Ask health questions anytime...
Feature description: Default slogan in the dialogue box.

Figure 2
对话: Dialogue
Feature description: Switch to Chatbot mode interface interface.

我的: PHR
Feature description: PHR interface.

晚上好: Good evening
从今天起做自己健康第一责任人: Be the first person accountable for your own health from today.
Feature description: Slogan.

本人>: Self >.
Feature description: Select PHR owner. iflyhealth app allows users to add multiple PHRs and select from the saved records for appropriate chats.

健康指标: Health Index
Feature description: Access PHR overview.

用药计划: Medication Plan
Feature description: A suggested personal medication plan developed based on saved PHR data.

复查计划: Follow-up Plan
Feature description: A suggested personal follow-up plan developed based on saved PHR data.

饮食建议: Dietary Recommendations
Feature description: Personal dietary recommendations generated based on saved PHR data.

运动建议: Exercise Recommendations
Feature description: Personal exercise recommendations generated based on saved PHR data.

健康史: Health History
既往史、个人史、家族史: Past History, Personal History, Family History
Feature description: Access Health History page for entering PHR history data.

资料夹: File Folder
累计帮你储存 0 份资料: Saved 0 files for you
Feature description: Total count of personal health files uploaded and saved on the app.

上传: Upload
Feature description: Upload file.

病历: Medical Record
共 0 份: 0 record

Feature description: Collection of uploaded medical record files.

体检报告: Health Checkup Report
共 0 份: 0 record
Feature description: Collection of uploaded health checkup report files.

报告单: Lab and Exam Reports
共 0 份: 0 record
Feature description: Collection of uploaded laboratory testing and examination report files.

未分类: Miscellaneous
共 0 份: 0 record
Feature description: Collection of other uploaded health files.

药物: Medications
共 0 份: 0 record
Feature description: Collection of saved medication data.

此健康信息已进行 数据安全保护: The current health information has been protected with data security.
Feature description: Access Data Security Policy.

Figure 3
健康史: Health History
Feature description: Name of current interface.

既往史: Past History
编辑: Edit
既往疾病: Past Disease
无: None
2024-11-11
手术情况: Surgery
无: None
2024-11-11
食物、药物等过敏情况: Food, Medication, and Other Allergies
无: None
2024-11-11
疫苗接种史: Vaccinations and Immunization History
无: None
Feature description: Enter and edit past history data in PHR.

个人史: Personal History
编辑: Edit
饮酒情况: Alcohol Consumption
无: None
2024-11-11
吸烟情况: Smoking History
无: None
2024-11-11
Feature description: Enter and edit personal history data in PHR.

家族史: Familial History
编辑: Edit
2 型糖尿病 妈妈: Type 2 diabetes, mother
2024-11-11
Feature description: Enter and edit familial history data in PHR. Note that the "Type 2 diabetes, mother" entry was made as an example to demonstrate the interface after data entry. No personal data breach risks are involved.
