# Supplementary material for: Harnessing an Artificial Intelligence–Based Large Language Model With Personal Health Record Capability for Personalized Information Support in Postsurgery Myocardial Infarction: Descriptive Qualitative Study
Source: J Med Internet Res. 2025 Apr 30;27:e68762. doi: 10.2196/68762 (PMC12079068; doi:10.2196/68762)
Supplement: Multimedia Appendix 2 [file jmir_v27i1e68762_app2.pdf]

Appendix 2: Interview Guide (Translated using ChatGPT)

Part 1: Initial Exposure and First Impressions

Early Awareness and Willingness to Use

- o How did you first learn about the iFlyHealth App?
- o What were your initial thoughts or expectations about the iFlyHealth App when you first heard about it?
- o Did your previous experience with other similar digital health tools (if any) influence your decision to try the iFlyHealth App?

Probing Questions:

- o What specific factors led you to decide to try (or not try) the iFlyHealth App initially?
- o Did anyone (such as family members or medical staff) influence your decision to use it?

First-Time Use Experience

- o How did you feel about setting up the iFlyHealth App and using its functions for the first time?
- o Were there any aspects of the initial setup or learning process that were particularly clear or confusing to you?

Probing Questions:

- o Did you receive any help from family members or medical staff during the initial setup or use?
- o Do you have any suggestions for improving the app registration process?

Part 2: Usage Patterns and Experience

Usage Frequency and Patterns

- o How often did you use the iFlyHealth App after your surgery?
- o Can you describe the situations or health issues that prompted you to use the iFlyHealth App?

Probing Questions:

- o Did you use it daily, or only when you had specific questions?
- o Has your usage frequency changed over time?

User Engagement

- o Which aspects of the app did you find most attractive or useful?
- o Were there any features that you found less useful or difficult to operate?

Probing Questions:

- After entering your personal health information, did you notice any changes in the relevance of the app's responses and suggestions to your situation?
- How would you evaluate the app's responses or suggestions regarding changes in your symptoms or health status?

Part 3: Perceived Benefits and Impact on Post-Operative Recovery

Access to Health Information

- How did the iFlyHealth App affect your ability to access health information related to your recovery?
- In what ways did the personalized information provided by the app impact you?

Probing Questions:

- Can you provide an example of when the app's information was particularly helpful to you?
- How did using the app influence your decisions regarding medication, lifestyle, or symptom management?

Comparison with Traditional Information Channels

- How does the iFlyHealth App compare with other health information sources you have used in the past (such as consulting doctors, brochures, or the Internet)?
- Do you feel more confident in managing your recovery process with the support of the app? Why or why not?

Probing Questions:

- In what aspects do you think the iFlyHealth App is superior to traditional channels?
- In what aspects do you think traditional channels are more reliable or helpful?

Part 4: Challenges, Barriers, and Reasons for Non-Use or Discontinuation

Technical Knowledge and Usability

- What challenges (if any) did you encounter while using the iFlyHealth App?
- How comfortable did you feel when using the app and asking questions to the AI?

Probing Questions:

- Did you need help from family members or medical staff while using the app?
- Do you have any suggestions to make the app more user-friendly?

Privacy and Security Concerns

- Were you concerned about the security or privacy of your personal health information while using the iFlyHealth App?
- Did these concerns affect your willingness to enter detailed health data?

Probing Questions:

- What information are you willing to share, and what information would you prefer to keep private?
- Do you think the app provided sufficient information regarding data security?

Reasons for Non-Use or Discontinuation

For participants who chose not to use the app or stopped using it later, understand the reasons behind this decision:

- Were there any specific features or aspects of the app that you felt were irrelevant to your recovery needs?

Probing Questions:

- Did issues such as outdated mobile devices, complexity of use, or insufficient personalization of responses affect your decision to use the app?
- What improvements do you think could encourage you to continue using the app?

Part 5: Reflections and Future Use Intentions

Overall Satisfaction and Impact

- Looking back, how would you summarize your overall experience with the iFlyHealth App?
- In what ways did the app impact your recovery process?

Probing Questions:

- Would you recommend the iFlyHealth App to other patients? Why or why not?
- How do you think this type of technology should evolve to better meet your needs?

Suggestions for Improvement

- What improvements or new features would you like to see in future versions of the iFlyHealth App?
- How could the app be improved to better address the challenges you experienced?

Probing Questions:

- Which features do you think are essential?
- What specific aspects need further optimization?

Closing

Final Thoughts:

"Is there anything else you would like to share about your experience with the iFlyHealth App or similar tools?"

Thank You and Next Steps:

"Thank you for your valuable time and feedback. Your insights will help improve the digital health experience for future patients and support the recovery process after myocardial infarction."

Note: Interviewers are not required to use all the questions. They should select and adapt questions based on the actual progress of the interview.

附录 2：访谈提纲

第一部分：初次接触与初步印象

早期了解与使用意愿

- 请问您最初是如何了解到讯飞晓医 App 的？
- 您了解到该讯飞晓医 App 时，您对它最初有哪些想法或者预期？
- （如果有)您之前使用其它类似数字医疗保健工具的经历是否对您尝试使用讯飞晓医的决定有影响？

探索问题：

- “具体是什么因素让您最初决定尝试（或者不尝试）使用讯飞晓医 App 的呢？”
- “有没有谁（比如您的家庭成员、医务人员等）对您的使用决定产生了影响？”

首次使用体验

- 您第一次设置讯飞晓医 App，使用这个 App 功能的感受是怎样的呢？
- 在第一次设置或学习使用过程中，是否有哪些方面您感觉特别清晰或着特别搞不明白的呢？

探索问题：

- “在第一次设置或者使用的过程中，您是否得到了家人或医务人员的帮助？”
- “您有没有什么可以改善 App 注册过程的建议吗？”

第二部分：使用模式与体验

使用频率与使用模式

- 您手术后，多长时间使用一次讯飞晓医 App？
- 您能否讲一讲有哪些情况或者健康问题，会促使您使用讯飞晓医 App？

探索问题：

- “您是每天使用，还是只有在遇到具体问题时才使用呢？”
- “您使用的频率随着时间有没有发生什么改变？”

用户参与度

- 您认为这个 App 哪些方面对您最有吸引力或者最有用？
- 有没有哪些功能您觉得不太有用或难以操作的？

探索问题：

- “在录入个人健康信息后，您是否注意到 App 的回答和建议跟您的情况之间的相关性发生了变化？”
- “您对讯飞晓医 App 关于症状或健康状况变化的回答或者建议有什么评价？”

第三部分：感受到的收获和对术后康复的影响

健康信息的获得

- 您感觉讯飞晓医 App 对您获取与康复相关健康信息的能力有什么作用吗？
- 您感觉 App 提供的个性化信息对您产生了哪些影响？

探索问题：

- “您能否提供一个对您特别有帮助的例子？”
- “使用这个 App 对您关于药物、生活方式或症状管理的决策有哪些影响呢？”

与传统信息渠道比较

- 讯飞晓医 App 与其他您以前用过的获取健康信息渠道（例如向医生咨询、宣传册、互联网等）相比如何？
- 您是否觉得在讯飞晓医 App 的支持下，对您的康复过程更有信心了？为什么？

探索问题：

- “您觉得讯飞晓医 App 在哪些方面优于传统渠道？”
- “在哪些方面您觉得传统渠道更可靠或者更有帮助？”

第四部分：困难、障碍及停止使用的原因

技术知识与易用性

- 您在使用讯飞晓医 App 过程中，曾经遇到过哪些挑战（如果有）？
- 您感觉使用讯飞晓医 App 和向人工智能提问题的过程舒适顺利吗？

探索问题：

- “您使用该讯飞晓医 App 过程中，需不需要家人或者医务人员的帮助？”
- “您有没有什么建议，帮助让这个 App 更好用？”

隐私与安全问题

- 在使用该讯飞晓医 App 时，您是否担心您的个人健康信息存在什么安全或隐私问题？
- 这些担忧对您录入详细健康数据的意愿有没有产生什么影响？

探索问题：

- “哪些信息您愿意分享，哪些信息您更愿意保密？”
- “您觉得讯飞晓医 App 是否为您提供的数据安全相关说明是否足够？”

不使用或停止使用的原因

对于选择不使用或者后期停止使用 App 的受访者，了解其做出这一决定的原因：

- 您觉得有没有设么具体的功能，或者 App 的哪些方面，跟您的康复需求没有关系？

探索问题：

- “手机太老、使用太复杂，或者回答的个性化不够等问题，会不会影响您使用 App 的决定？”
- “您觉得有哪些可以改善的地方可以让您您继续使用？”

第五部分：反思与未来使用意愿

总体满意度与影响

- 现在回忆起来，您觉得您对讯飞晓医 App 的整体使用体验如何？
- 这个 App 在哪些方面影响了您的康复过程？

探索问题：

- “您是否会向其他病友推荐讯飞晓医 App？推荐的理由？”
- “您觉得这类技术未来如何发展才能更好地满足您的需求？”

改进建议

- 您希望在讯飞晓医 App 未来的版本里面，看到哪些改进或者新增的功能？
- 讯飞晓医 App 可以如何改进，才能更好地解决您的问题？

探索问题：

- “您觉得哪些功能是必不可少的？”
- “具体哪些方面需要进一步优化？”

结束语

最后想法：

“您还有没有其他关于讯飞晓医 App 或者类似工具的使用体验想要补充，或者跟我再分享一下？”

感谢与后续：

“感谢您抽出宝贵的时间，提供宝贵反馈。您的反馈将有助于改善未来患者数字健康体验，支持心梗后的康复过程。”

注：访谈者不需要使用所有问题，应根据访谈实际进展选择、调整问题。
